# An evaluation of the elements of internal medicine physiopathology curriculum in general practice based on the perspectives of faculty members of Shiraz University of Medical Sciences

**Published:** 2015-04

**Authors:** JAMSHID ESLAMI, MOHSEN KHADEMI

**Affiliations:** Department of Curriculum Planning, Educational and Psychological Sciences Faculty, Shiraz University, Shiraz, Iran;

**Keywords:** Evaluation, Curriculum, Faculty

## Abstract

**Introduction:**

An evaluation of the curriculum elements can be recognized as a necessity in curriculum dynamic and improvement. This study aimed at evaluating five main elements of a physiopathology curriculum in internal medicine (objectives, content, methods, evaluation, and management).

**Method:**

The present study is of a descriptive-analytical type, and the studypopulation consisted of a total of 48 faculty members of internal medicine physiopathology departmentat Shiraz University of Medical Sciences. Participants wereselected using Cochran’s sample size formula andthrough simple random sampling.Thedatawere collected using a 58-item questionnaire devised by the researcher, usingcurriculum planning experts. Face and content validity of the scale were obtained throughexpert views and modifications provided by 10 professors and experts in medical curriculum evaluation. Also, research reliability was calculated using Alpha Cronbachto be 0.99. Reliability value and coefficient was acceptable.Moreover, One-sample t-test, Independent t-test and one-way ANOVA were used for data analysis.

**Results:**

Based on the faculty members’ views, of the five curriculum elements, objectives and content were in relatively good conditions (at an average level) while other elements including method, evaluation and management were in poor conditions (lower than average). According to results oftwo-way ANOVA, there wasa significant relationship between faculty members with various work experiencein terms of curriculum evaluation.

**Conclusion:**

According to research findings, a comparative examination of the curriculum elements and their characteristics in physiopathology course can be conducted, resulting in identification of curriculum weaknesses and their pitfalls. Also, with regard to teaching, evaluation, management methods, weak and strong pointsof the course,efficiency, and effectiveness of the elements were identified.

## Introduction

Nowadays, quality is of high significance in universities and medical higher education institutions, and its improvement has remained as one of their main concerns. Identifying weak and strong points, recognizing threats, and opportunities, and also making attempts to promote the status quo, and to achieve perfect and optimal conditions are of their main duties. Continuous quality improvement requires continuous evaluation. Curriculum evaluation is of the methods having high efficiency in continuous improvementof academic systems and is also considered as the most significant step in establishing quality systems. It refers to a formal activity undertaken with the aim of determining the degree of quality, effectiveness, and value of curriculum elements. Therefore, every educational system requires it if it seeks to be efficient in achieving its objectives. With regard to increasing improvements and changes occurringin educational status and needs at the global and national level, the necessity of revising higher education curriculum seems inevitable. To achieve this goal, a comprehensive evaluation of the curriculum in terms of all of its parts and factors is required (1). Curriculum is the heart of education, the revision of which in different fields has attracted the attention of educational evaluators. Revising university curriculum is a continuous, vital, and inevitable phenomenon (2). It is curriculum evaluation that shows to what extend each one of the elements are in line and performable with regard to learner conditions and other facilities and restrictions (3). Curriculum can never be considered as complete, and due to permanent changes and improvements of its elements, its revision is of the undeniable requirements if it is supposed to be dynamic and improved(4). In addition, curriculum evaluation leads to more precise knowledge of learning goals, educational activities, results, and outputs (5). Either formally or informally, and in the form of a momentary decision-making, evaluation is a philosophical process which seeks to properly value a certain person, process, and an object (6). Keating (2011) believes that evaluation is a process utilized to collect data to determine the final value of an educational phenomenon (7). According to Nitko (2001), evaluation is a process judging the quality of knowledge, skills, and capabilities that learners are expected to acquire after education (8). In fact, it is an attempt made to estimate the quality of plans and services against standards and to make sure that they are in line with each other (9). As put by Bennet: “approving quality in higher education without its evaluation is impossible” (10). Responding to challenges of medical education in the third millennium, and achieving goals of 1404 Vision Policy require improving educational quality in universities, medical higher education institutions, and different courses. Today that we are facing rapid growth in medical education courses all over the country, by adopting appropriate strategies, we should not only be aware of the current status of medical education system in the country, but also pave the way for enhancing the effectiveness of these courses so that the society demands can be met. Therefore, the need for existence of an evaluation pattern and framework is felt, especially in terms of examining improvements in the quality of curriculum elements. Training efficient and effective doctors has always been taken into account by medical faculties. It requires examining educational program and curriculum, and also its revision and quality improvement (11). Thus, medical sciences curriculum, like that of other majors, needs precise evaluation in order for it to be qualitatively improved. The curriculum of physiopathology courses in internal medicine is one of these programs. Physiopathology course, the second phase of internal medicine, or clinical introduction is a one-year course aiming at preparing students for entering theclinical internship. By evaluating different elements of physiopathology curriculum, its efficiency and effectiveness can be increased. A comprehensive and clear evaluation of the elements of physiopathology courses, their repetitive revision, and conducting necessary chapter modifications based on the needs of society and students, and also updated scientific changes and advances can lead to continuous improvements in the quality of the educational courses. Moreover, direct contribution of faculty members in evaluation, and surveying opinions of those concerned with and interested in educational courses, as it is sought by the present study, will result in fostering accountability culture, continuous improvement in the course quality, and optimal education. Many studies are conducted on evaluation of the quality of internal medicine curriculum, usingthe world various patterns including.

According to Akhlaghi et al.(12), internal evaluation of curriculum showed low budget, facility and equipment of educational course. Moreover, curriculum was not in line with approved chapters, course prerequisites, students’ occupational interests and capabilities, students’ expectations and needs, and advances in science and technology. Based on national standards, Khajezad (13) found that the quality of internal medicine curriculum is reported to be good and poor by professors and students, respectively. Moreover, according to standards of World Federation for Medical Education, he figured out that the quality of internal medicine curriculum is reported to be acceptable and lower than acceptable level by professors and students, respectively. Results of a study conducted by Farzianpour et al.(14) showed that the basic standards of WFME pattern have relatively been observed in Tehran University of Medical Sciences (73%). Also, according to Shekarchi et al.(15), the basic standards of WFME pattern have been observed in all the evaluation components of the university, and that curriculum is in a good condition. Moreover, Adalatkhah et al.(16) found that objectives and contents of medical curriculum do not satisfy the students’ needs, and they call for changes in many elements of the program. In addition, Sunyadi andKudwadi (17) found that evaluation can provide curriculum developers with an appropriate vision with regard to attempts for curriculum modification and improvement. According to Kligler et al.(18), in case internal evaluation coincides with other validation stages like external evaluation, professional counterparts’ visit to the site, and peer assessment, it will have its effect on planning process for improvements at different levels. Moreover, Cohen and Jocobs (2004) (19) found that continuous evaluation is a necessity for the survival of a competing and safe curriculum. They also concluded that none of the composing parts of a curriculum should be considered as unimportant. Also, according to Regenstreif et al. (2004) (20), basically, to be more effective, internal evaluation needs various qualitative methods including mentoring experts. Also, according to Bazrafkan et al., lesson plans in 94% of cases have been effective in curriculum. On the other hand, the significant factor has been flexibility. Also, since professors have contributed tolesson plans, the curriculum can be developed according to goals (21).

## Methods

This research is of a descriptive-analytical type; the statistical population was composed of 48skilled faculty members of internal medicine physiopathology course at Shiraz University of Medical Sciences who had experience in medical curriculum and its evaluation. A total of 48 out of 55 members were selected using Cochran’s sample size formulaandthrough simple random sampling. Research data were collected using a 58-item questionnaire devised by the researcher who made use of curriculum planning experts. The questionnaire evaluated five physiopathology curriculum elements (objective, content, method, evaluation, and management).Both face and content validity of the scale were obtained utilizing expert views, and modifications were provided by 10 professors and experts in medical curriculum evaluation. Also, research reliability was calculated using Alpha Cronbach (0.99). In order to analyze the data and also evaluate the status of Shiraz University of Medical Sciences Physiopathology curriculum in general and based on five elements, weusedone-sample t-test. Moreover, in order to determine whether faculty members’ practical experience in evaluation of physiopathology curriculum and their previous record has influenced their curriculum evaluation and also in order to determine the effect of faculty members’ gender on their evaluation of physiopathology curriculum,two-way ANOVA and independent t-test were used, respectively.

## Results

In order to evaluate the quality five elements of physiopathology curriculum (objective, content, method, evaluation, and management) based on evaluation criteria (appropriateness, coherence, and balance), which are incorporated into the scale, and views of the medical faculty members, and also to determine to what extent qualitative components are observed in the curriculum elements, one-sample t-test was utilized. Then, the views of the faculty members were compared to the criterion score (score 3), which is the average score concerning every element and 5-point Likert scale(Table1).

**Table 1:** Results of the one-sample t-test in the examination of the faculty members’views on the quality of five curriculum elements

**Criterion Score (3)**					
**Curriculum elements**	**Num**	**Mean±SD**	**Degree of freedom**	**t **	**Sig**
**Objective**	48	3.039±0.560	47	0.492	0.625
**Content**	48	2.856±0.587	47	-1.700	0.096
**Teaching method**	48	2.691±0.670	47	-3.189	0.003
**Management**	45	2.471±0.722	44	-4.902	0.0001
**Evaluation**	48	2.329±0.846	114	-5.494	0.0001

Results of the analysis showed that there was not a significant difference between the criterion score and score of the objective element obtained from the questionnaire (p>0.050). Faculty members’ evaluation mean score of the objective quality (3.039) was higher than the criterion mean score (3), but the difference wasnot significant. Therefore, according to the faculty members, the objective element has an average status (close to mean) with regard to qualitative components of the questionnaire. Considering content element, no significant difference was reported (p>0.050). Faculty members’ evaluation mean score regarding the quality of content element (2.856) waslower than the criterion mean score (3), but this difference is not also significant. Thus, the content element has an average status (close to mean) with regard to observing qualitative components of the questionnaire. But, with regard to three other elements (method, management, and evaluation), faculty members’ evaluation mean score waslower than criterion mean score (3), resulting in a significant difference (p<0.050).Accordingly, the latter elements are in a poor condition (lower than average).

Also, to examine the interactive effect of faculty members’ workhistory and their practical experience in curriculum evaluation on their evaluation of physiopathology intended curriculum, we used Two-way ANOVA (Table 2).

**Table 2:** Results of two-way ANOVA for examining the interactive effect of faculty members’ Service History and practical experience on their evaluation of physiopathology curriculum

**Source of variance**	Degree of freedom	Mean	F	Sig
**Service History**	2	32.099	3.485	0.041
**Practical experience in evaluation**	2	20.732	2.251	0.119
**Service History × practical experience in evaluation**	1	4.730	0.514	0.478
**Intra-group**	39	9.210		
**Total**	44			

Two-way ANOVA is a method which analyzes simple and interactive effects of two or more independent variables as a dependent variable. In other words, independently of or interacting with each other, two or more independent variables change in this analysis to build changeability of the dependent variable. The main effect in variance analysis is the effect of each one of the factors, irrespective of the effect of other factors. As an example, the effect of faculty members’ service history (by itself) on their evaluation of physiopathology intended curriculum is called the main effect while in interactive effect, in case two factors are taken into account simultaneously, it is called the interactive effect test.

In order to run variance analysis in the current research, firstly normality and equality of dependent variable variance is examined through Leven test. Considering that the significance level of calculated Leven value was higher than 0.05, research data did not question the equality assumptions of error variances, so allowing the possibility of using variance analysis. 

In order to conduct variance analysis, firstly assumptions concerning the analysis are presented. Then, with regard to F statistics and also significance level obtained from the analysis, the independent (main) and interactive effect of two variables (faculty members’ service history and their practical experience in curriculum evaluation) on their evaluation of physiopathology intended curriculum is reported.

**The null hypothesis of the main effect of service history**: Society means (the status of the evaluation of physiopathology intended curriculum) were equal among faculty members with differing service histories. That is, there is not a significant difference between faculty members with differing service histories in terms of evaluating physiopathology intended curriculum. According to results of the analysis, there was a significant difference between faculty members with differing service histories in terms of evaluating physiopathology intended curriculum (MS=32.099, F=3.485, p=0.041). These results showed that faculty members with differing service histories do not hold the same attitude towards physiopathology intended curriculum and that their evaluation of the status of the mentioned curriculum is different. Hence, null hypothesis is rejected. In addition, to determine among which groups the differences lie, we used Tukey test, the results of which showed that the differences lied in faculty members with service histories of 1-10 and 11-20 years. 

**The null hypothesis of the main effect of differing experiences in evaluation:**society means (the status of the evaluation of physiopathology intended curriculum) were equal among faculty members with differing experiences in curriculum evaluation. That is, there was nosignificant difference between faculty members with differentexperiences in curriculum evaluation in terms of evaluating physiopathology intended curriculum.

According to the results of the analysis, there wasnot a significant difference between faculty members with differing experiences in curriculum evaluation in terms of evaluating physiopathology intended curriculum (MS=20.732, F=2.251, p=0.119). These results show that faculty members with differentexperiences in curriculum evaluation held the same attitude towards physiopathology intended curriculum and that their evaluation of the status of the mentioned curriculum was the same. Hence, null hypothesis is proven.

**The null hypothesis of the interactive effect: **The effect of faculty members’ service history and their different experiences in curriculum evaluation on the dependent variable (the status of the evaluation of physiopathology intended curriculum) was the same. That is, there was no interactive effect between the two variables. 

As it is obvious, the interactive effect significance level was higher than 0.05, showing that faculty members’ service history and their different experiences in curriculum evaluation hadno interactive effect on their evaluation of physiopathology intended curriculum (MS=4.730, F=0.514, p=0.478). Therefore, the null hypothesis was proven. 

## Discussion

According to the findings of the present study, a significant difference between five curriculum elements has been shown with regard to curriculum standard characteristics. Two elements of objective and content hadan average status while other three elements were in completely poor condition qualitatively (method, management, and evaluation). In other words, physiopathology curriculum goals and content aresomehow based on standards and students’ and faculty members’ needs and characteristics, and also have temporal and spatial requirements. Thus, their evaluation is in poor conditions. Findings of the present study are in line with those of Edalatkhah et al. (2005)(16) and Akhlaghi et al. (2011)(12). They also addressed evaluation of medical education and the quality of educational programs in higher education centers based on medical graduates’ and interns’ views resulting in the conclusion that their quality is not in a promising condition while Farzianpour et al. (2008)(14) held that elements of medical curriculum have the necessary quality. Thus, according to researches similar results continuity of poor quality of medical curriculum elements can be taken into account. Due to the fact that none of the addressed elements has been qualitatively in a good condition, is the followings are suggested:

Revision of curriculum elements with regard to internal and external evaluation criteria (appropriateness, coherence, and balance), characteristics, conditions, and requirements of medical education in Iran’s cultural environment should be addressed. In order to achieve this goal, it is required that while setting curriculum objectives, aspects of individuals’ physical, mental, social, and spiritual health should be taken into account in terms of scientific basis and evidence, and also appropriateness of the objective with needs of individuals and society. In order to provide the ground for continuous learning, theoretical and practical objectives of the course should be congruent with each other. Also, content should change in accordance with objectives, and scientific achievements and human experiences be introduced in an Islamic framework. Size and content of textbooks should be compatible with students’ capabilities and characteristics, and lessons normal sequence should be maintained. Content should be related to society issues at local, regional, and national scales and have unity. It should also have a balanced look at different aspects of medical education. 

With regard to teaching method, different methods of theoretical, practical, interactive, and clinical teaching should be applied and cooperative and active learning experiences should be created. New management and planning strategies including cooperative management and strategic planning should be taken into account and also all the curriculum planning staff including interns should contribute tocurriculum planning and implementing. Different evaluation methods should be applied. 

## Conclusion

Results of the present study help the curriculum decision- and policy-making at the macro level. It also helps planners and officials in deciding about continuing, preventing or revising curriculum elements, their characteristics and educational processes. The study showednot only the curriculum status quo, its weak and strong points, but it also the professors and planners’future vision of physiopathology curriculum evaluation towards its localization and improvement stages. 

The present study had some limitations.Some of professors were unwilling and tendency to cooperate and complete the questionnaire precisely; there was no easy access to those at other departments, and also similar evaluation models were unavailabe, especially in physiopathology courses which posed problems for comparing the results and analyses. 
